# Functional Organization for Response Inhibition in the Right Inferior Frontal Cortex of Individual Human Brains

**DOI:** 10.1093/cercor/bhaa188

**Published:** 2020-07-15

**Authors:** Akimitsu Suda, Takahiro Osada, Akitoshi Ogawa, Masaki Tanaka, Koji Kamagata, Shigeki Aoki, Nobutaka Hattori, Seiki Konishi

**Affiliations:** 1 Department of Neurophysiology, Juntendo University School of Medicine, Tokyo 113-8421, Japan; 2 Department of Neurology, Juntendo University School of Medicine, Tokyo 113-8421, Japan; 3 Department of Radiology, Juntendo University School of Medicine, Tokyo 113-8421, Japan; 4 Research Institute for Diseases of Old Age, Juntendo University School of Medicine, Tokyo 113-8421, Japan; 5 Sportology Center, Juntendo University School of Medicine, Tokyo 113-8421, Japan; 6 Advanced Research Institute for Health Science, Juntendo University School of Medicine, Tokyo 113-8421, Japan

**Keywords:** areal parcellation, boundary mapping, functional connectivity, inferior frontal gyrus, stop-signal task

## Abstract

The right inferior frontal cortex (IFC) is critical to response inhibition. The right IFC referred in the human studies of response inhibition is located in the posterior part of the inferior frontal gyrus and the surrounding regions and consists of multiple areas that implement distinct functions. Recent studies using resting-state functional connectivity have parcellated the cerebral cortex and revealed across-subject variability of parcel-based cerebrocortical networks. However, how the right IFC of individual brains is functionally organized and what functional properties the IFC parcels possess regarding response inhibition remain elusive. In the present functional magnetic resonance imaging study, precision functional mapping of individual human brains was adopted to the parcels in the right IFC to evaluate their functional properties related to response inhibition. The right IFC consisted of six modules or subsets of subregions, and the spatial organization of the modules varied considerably across subjects. Each module revealed unique characteristics of brain activity and its correlation to behavior related to response inhibition. These results provide updated functional features of the IFC and demonstrate the importance of individual-focused approaches in studying response inhibition in the right IFC.

## Introduction

Studies of neuropsychology, neuromodulation, and neuroimaging have highlighted the importance of the human right inferior frontal cortex (IFC) in response inhibition (Aron et al. 2004b). Damage to the right inferior frontal gyrus in the IFC (Aron et al. 2003), specifically the pars opercularis (Aron et al. 2004a), results in prolonged stop-signal reaction time (SSRT), which is a behavioral measure to evaluate response inhibition performance in the stop-signal task. Transcranial magnetic stimulation to the pars opercularis in the inferior frontal gyrus also prolonged the SSRT (Chambers et al. 2006). On the other hand, neuroimaging studies have reported brain activation during response inhibition in wider regions in the right IFC, including the pars opercularis, pars triangularis, inferior frontal junction (IFJ), and ventral part of the precentral sulcus (Hirose et al. 2009; Swick et al. 2011; Aron et al. 2014). A correlation between the brain activity and SSRT has also been reported in these regions in the right IFC (Aron et al. 2007; Congdon et al. 2010; Jimura et al. 2014). Moreover, subregions in the right IFC have been demonstrated to implement distinct functions related to response inhibition. For example, the ventral part of the IFC is more critical to updating action plans, whereas the dorsal part of the IFC is involved in the visual detection of changes in the environment (Chikazoe et al. 2009; Verbruggen et al. 2010). These prior studies highlight the need for a more comprehensive and precise understanding of the areal organization of the right IFC.

Functional connectivity can be used to parcellate the cerebral cortex into subregions (cortical parcels) that approximate functional areas (Mars et al. 2012; Wig et al. 2014b; Eickhoff et al. 2018; Van Essen et al. 2019). Moreover, precision functional mapping focusing on single subjects has been advocated as a model for neuroimaging studies examining the organization of healthy and diseased individual human brains (Finn et al. 2015; Laumann et al. 2015; Xu et al. 2016; Braga and Buckner 2017; Gordon et al. 2017b; Noble et al. 2017; Gratton et al. 2018; Kong et al. 2019; Greene et al. 2020; see Gratton et al. 2020 for review). In the present study, for a more comprehensive and precise understanding of the areal organization of the right IFC at the single-subject level, we parcellated the right IFC in individual brains and evaluated the functional properties related to brain activation and brain-behavior correlation during response inhibition. The IFC was defined in the present study as the pars opercularis and its surrounding regions in the frontal cortex (the pars triangularis, IFJ, and precentral sulcus). Based on group data, six parcels were identified in the right IFC (Fujimoto et al. 2020). Parcels in the right IFC of individual subjects were classified into the six “modules” or subsets of parcels (Bullmore and Sporns 2009; Nelson et al. 2010), which were defined by the spatial pattern of the group-level parcel-cortex functional connectivity. The functional properties of each module were then evaluated in terms of the brain activation and its correlation with SSRT during the performance of the stop-signal task at the single-subject level.

## Materials and Methods

### Subjects

Twenty right-handed subjects (10 men and 10 women, age: 26.6 ± 9.2 years [mean ± SD]) participated in the experiments. Written informed consent was obtained from all subjects according to the Declaration of Helsinki. The experimental procedures were approved by the Institutional Review Board of Juntendo University School of Medicine.

### Magnetic resonance imaging Procedures

Image data were acquired using a 3-T magnetic resonance imaging (MRI) scanner and a 64-channel radio frequency head coil (Siemens Prisma, Germany). T1- and T2-weighted structural images were first obtained (resolution = 0.8 × 0.8 × 0.8 mm^3^). Functional images were then obtained using multiband gradient-echo echo-planar sequences (TR = 1.0 s, TE = 30 ms, flip angle = 62°, FOV = 192 × 192 mm^2^, matrix size = 96 × 96, 78 contiguous slices, voxel size = 2.0 × 2.0 × 2.0 mm^3^, multiband factor = 6, phase-encoding direction = posterior to anterior). In the resting-state scan (Osada et al. 2017, 2019; Ogawa et al. 2018; Tamura et al. 2019; Fujimoto et al. 2020; Tanaka et al. 2020), subjects were asked to fixate on a cross displayed on a screen. One run consisted of 360 volumes, and 10 runs were administered. In the task scan, subjects performed the stop-signal task (see Behavioral Procedures). One run consisted of 330 volumes, and 10 runs were administered. Before each run, one functional image was acquired with the opposite phase-encoding direction for subsequent topup distortion correction in the image preprocessing.

### Areal Parcellation Procedures

For the resting-state dataset, preprocessing was conducted mainly following Human Connectome Project (HCP) Pipelines (Glasser et al. 2013, 2016). Functional images were realigned, topup distortion corrected (Andersson et al. 2003), and spatially normalized to the Montreal Neurological Institute (MNI) template. Time-series data were cleaned using the ICA-FIX method (Salimi-Khorshidi et al. 2014). After volumetric preprocessing, the image data were projected to 32k fs_LR surface space using the full version of the multimodal surface matching method (MSMAll) (Robinson et al. 2014; Glasser et al. 2016). We evaluated the amount of head motion by employing frame-wise displacement (Power et al. 2012), a measurement of instantaneous head motion that can be calculated as a locational difference between two successive volumes. Frames with frame-wise displacement > 0.25 mm as well as uncensored segments of data lasting fewer than 5 contiguous volumes were censored; all such data were excluded from the subsequent parcellation analysis.

The parcellation analyses based on boundary mapping (Margulies et al. 2007; Cohen et al. 2008; Biswal et al. 2010; Zhang et al. 2012; Zhang and Li 2012; Hirose et al. 2012b, 2013, 2016; Wig et al. 2014a; Laumann et al. 2015; Poldrack et al. 2015; Wang et al. 2015; Glasser et al. 2016; Gordon et al. 2016, 2017b; Osada et al. 2017, 2019; Ogawa et al. 2018; Fujimoto et al. 2020) were applied to the cerebral cortical surface. The mean cortical grayordinate signal was regressed out, followed by spatial smoothing (FWHM = 6 mm). Each vertex in the cortical surface of each subject was used as a seed to calculate its correlations with all the vertices. The spatial pattern similarities of the correlation maps were evaluated using correlation coefficients (similarity maps). Spatial gradients of the similarity maps were computed for each seed vertex. After spatial smoothing (FWHM = 6 mm), a two-dimensional watershed algorithm was applied to the smoothed gradient maps, and the binary watershed maps were averaged across seed vertices to generate a boundary probability map. The watershed algorithm was again applied to the boundary probability map to delineate parcellated regions (parcels) for each subject. For a group analysis, the gradient maps were averaged across the subjects, and the same procedures (spatial smoothing and watershed application) were applied to the averaged gradient maps. There were 611.8 ± 18.2 (mean ± SD) parcels identified in the cortical surfaces in individual brains, and 377 parcels identified in the group analysis, consistent with the results of previous studies of cortical parcellation (Bellec et al. 2010; Craddock et al. 2012; Shen et al. 2013; Finn et al. 2015; Laumann et al. 2015; Glasser et al. 2016; Gordon et al. 2016, 2017b; Schaefer et al. 2018).

To avoid a nonuniform signal-to-noise ratio caused by the different number of vertices in the parcels, we defined a region of interest (ROI) for each parcel for subsequent analyses. The size of the ROI was 30 vertices for a single subject analysis and 40 vertices for a group analysis that were closest to the centroid of the parcel. When the parcel contained less than these numbers of vertices, the ROI included all of the vertices in the parcel.

### Modules in the Right IFC of Individual Brains

**Figure 1:**
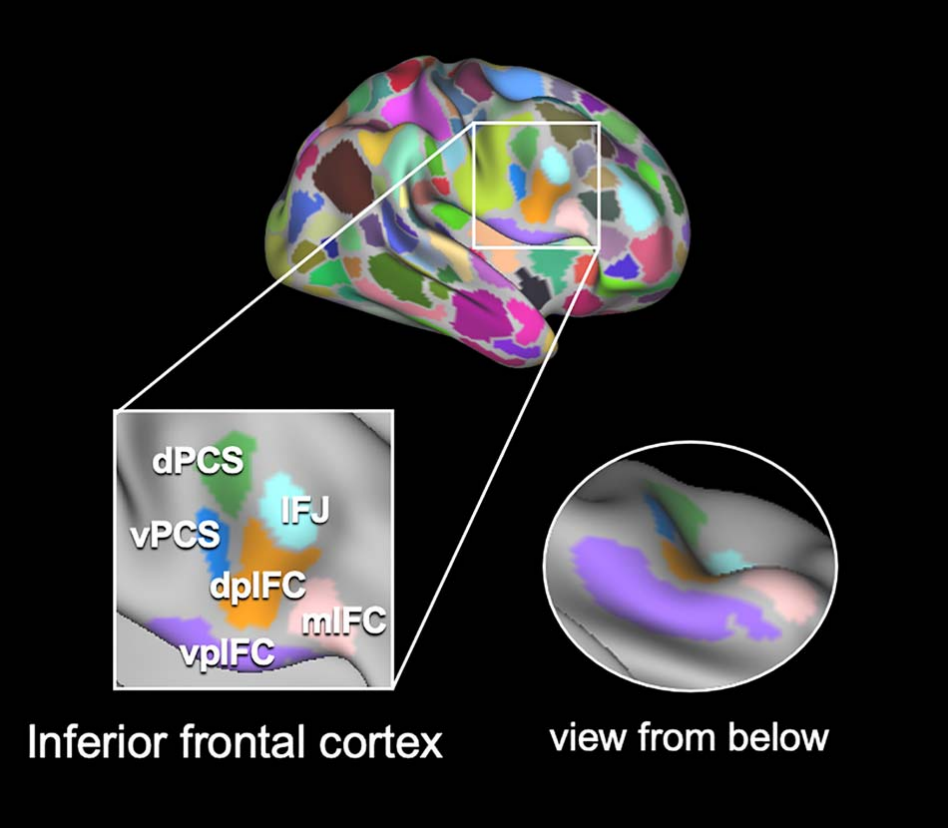
Parcels in the right IFC in group data. There were six parcels in the right IFC in the group data: the vpIFC, shown in purple; dpIFC, shown in orange; IFJ, shown in light blue; mIFC, shown in pink; vPCS, shown in blue; and dPCS, shown in green. These parcels were located in the pars opercularis, which has been shown to be critical to response inhibition, and its surrounding regions in the frontal cortex (the pars triangularis, IFJ, and precentral sulcus).

The IFC was defined in the present study as the pars opercularis, which has been shown to be critical to response inhibition, and its surrounding regions in the frontal cortex (the pars triangularis, IFJ, and precentral sulcus). The right IFC consisted of six parcels at the group level: the ventral posterior IFC (vpIFC), dorsal posterior IFC (dpIFC), IFJ, middle IFC (mIFC), ventral precentral sulcus (vPCS), and dorsal precentral sulcus (dPCS) (Fig. 1; see Supplementary Table 1 for MNI coordinates of the centroid of each parcel). The coordinates indicate that the IFJ in the present study corresponds to the same IFJ in previous studies (Derrfuss et al. 2005). Note that the anterior bank of the precentral sulcus is located in the vpIFC and dpIFC, whereas the posterior bank of the precentral sulcus is located in the vPCS and dPCS. The vpIFC corresponds to FOP1 in Glasser et al. (2016) and CinguloOperc_38 in Gordon et al. (2016). The dpIFC corresponds to area 6r and the rostral part of DorsalAttn_32. The IFJ corresponds to IFJp and the dorsal part of DorsalAttn_32. The mIFC corresponds to area 44/45 and VentralAttn_18. The vPCS corresponds to area 6v and the caudal part of DorsalAttn_32. The dPCS corresponds to PEF and DorsalAttn_31 (see also Supplementary Table 2 for other classifications).

**Figure 2:**
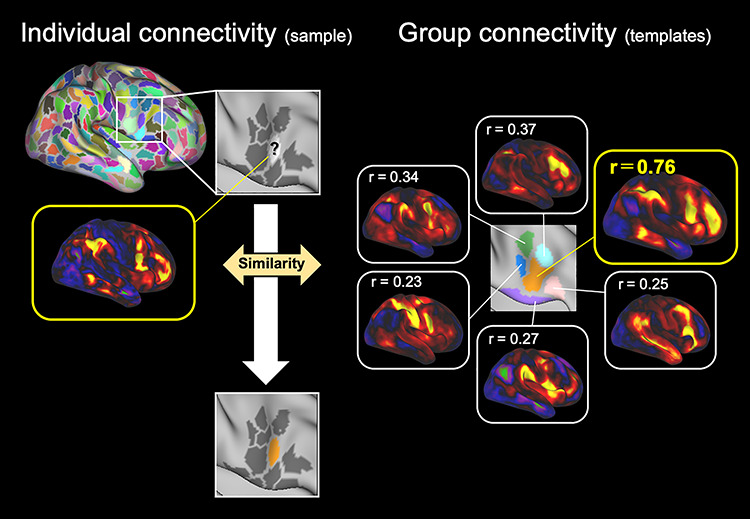
Assignment of IFC parcels of individual brains to one of six modules. The parcels in the right IFC of individual subjects were assigned to the six modules based on the spatial similarity between the cortical correlation maps for the IFC parcels of individual subjects (left) and the six cortical correlation maps in the group data (right). The dpIFC, shown in orange, had the most similar connectivity pattern, shown in a yellow line.

The functional connectivity between each of the six parcels in the right IFC, and the whole cerebral cortex was calculated to generate cortical correlation maps for the six parcels in the group data (Fig. 2). The parcel-cortex functional connectivity was also calculated for individual brains to generate cortical correlation maps for the IFC parcels. Parcels in the right IFC of individual subjects were classified into the six “modules” or subsets of parcels (Bullmore and Sporns 2009; Nelson et al. 2010; Miyamoto et al. 2013), which were defined by the spatial pattern of the group-level parcel-cortex functional connectivity. Each right IFC parcel of individual brains was assigned to one of the six modules that had the most similar connectivity pattern, that is, the spatial similarity between the cortical correlation map for each IFC parcel of individual subjects and the six cortical correlation maps in the group data (Fig. 2). When the similarity of the parcels did not reach the threshold of *r* (correlation coefficient) = 0.5 in any of the six connectivity patterns, the parcels were not assigned to any of the six modules.

### Behavioral Procedures

In the task scan, the same subjects performed the stop-signal task (Aron et al. 2003; Osada et al. 2019). The task procedures conform to the guidelines described in Verbruggen et al. (2019). The stop-signal task comprised of Go trials and Stop trials. At the beginning of a trial, a circle was presented for 500 ms at the center of the screen as a warning. In Go trials, a left- or right-pointing arrow (Go signal) was presented inside the circle, and the subjects were instructed to press a button indicating the corresponding direction with their right thumb. In Stop trials, the Go signal was first presented inside the circle, similar to Go trials. After a stop-signal delay (SSD), however, the arrow was changed to an up-pointing arrow, and the subjects were required to withhold manual response. The SSD was updated with each Stop trial based on a tracking procedure with increments or decrements of 50 ms, enabling maintenance of the accuracy of Stop trials at approximately 50%. To evaluate the efficiency of the response inhibition, this study estimated the SSRT as a behavioral index for efficient response inhibition for each subject based on an integration method (Logan and Cowan 1984; Verbruggen et al. 2013). Prior to test runs, two practice runs were performed. Each run comprised of 96 Go trials and 32 Stop trials. To verify the independent race model in the stop-signal task, the following three assumptions were confirmed (Verbruggen et al. 2019): 1) reaction time in Stop failure trials is shorter than in Go success trials, 2) reaction time in Stop failure trials increases as a function of the SSD, and 3) the probability of Stop failure trials increases as a function of SSD.

### Image Analysis for Task Dataset

For the task dataset, preprocessing was conducted similarly to the resting-state dataset except for ICA-FIX denoising. The data were also projected to 32k fs_LR surface space. Task activation was analyzed in each vertex or on the basis of the parcels by averaging time-series signals across vertices. A general linear model was then applied to each vertex or each parcel using FSL (https://fsl.fmrib.ox.ac.uk/fsl/fslwiki). Two events of interest (Go success and Stop success), together with nuisance events (Go failure and Stop failure), were coded at the onset of the Go signal of each trial, and were modeled as transient events convolved with a canonical hemodynamic response function and its temporal derivative. Six parameters of head motion derived from realignment were also included in the model as covariates of no interest. Time-series data were filtered with a high-pass filter (cutoff: 64 s). The magnitude images for individual subjects were contrasted between Stop success and Go success trials.

## Results

**Figure 3:**
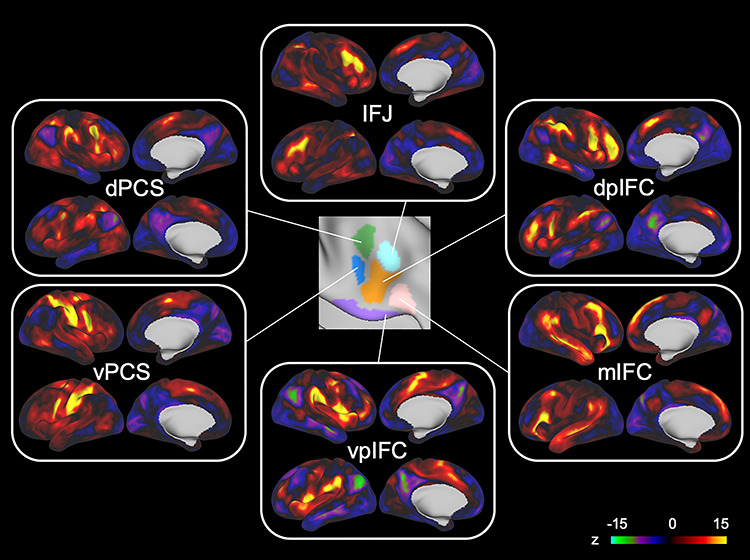
Cortical correlation maps for six modules in the group data. The cortical correlation maps were generated by calculating the functional connectivity between each of the six parcels in the right IFC and the whole cerebral cortex. The six parcels had distinct connectivity patterns with the cerebral cortex and were used for subsequent analyses to assign the IFC parcels of individual brains.

**Figure 4:**
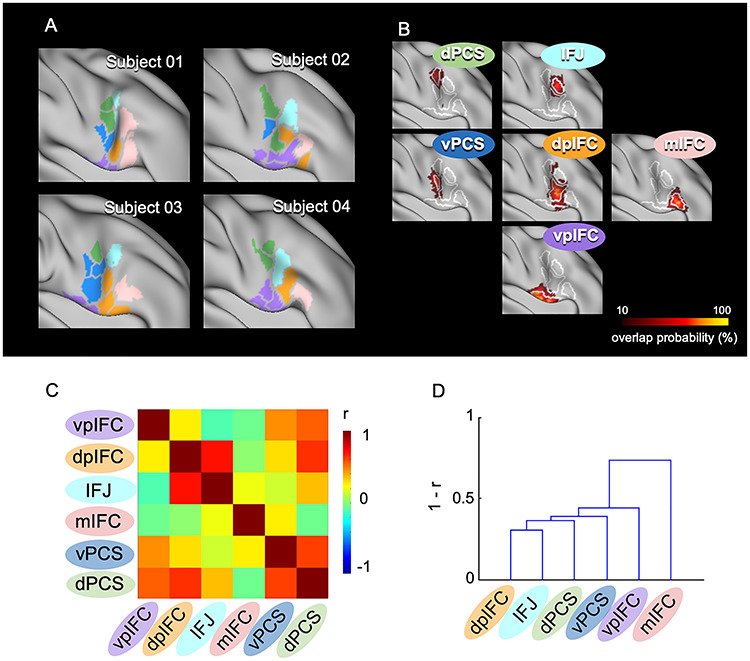
IFC parcels of individual brains assigned to six modules. (*A*) The right IFC parcels were assigned to the six modules in four representative subjects. The colors in the parcels indicate the six modules shown in Fig. 1, and the gray parcels indicate parcels not assigned to any of the six modules. (*B*) Vertex-wise probabilistic maps of the six modules in the right IFC averaged across subjects. (*C*) The matrix of similarity of the cortical correlation maps among the six modules, averaged across subjects. The color scale indicates the correlation coefficient. (*D*) The dendrogram of the similarity matrix among the six modules.

The right IFC parcels of individual brains were assigned to one of the six modules based on the six group-level cortical correlation maps shown in Figure 3. The module organization of the right IFC in four representative subjects are shown in Figure 4*A* (see Supplementary Fig. 1 for all the subjects). Although the overall organization of the six modules was largely maintained (Fig. 4*B*), there was variability in the spatial extent of the modules. Table 1 summarizes the proportion of the six modules in the IFC parcels of individual brains. The vpIFC and dpIFC modules were observed robustly in the right IFC, but the IFJ, mIFC, vPCS, and dPCS modules were absent in some subjects. Figure 4*C* shows the matrix of the similarity of the cortical correlation maps among the six modules, averaged across subjects. The dendrogram of the matrix indicates that, relative to other combinations, the dpIFC and IFJ modules exhibited higher similarity (Fig. 4*D*).

**Table 1:** Numbers and proportions of the IFC parcels for the six modules in each subject

	vpIFC	dpIFC	IFJ	mIFC	vPCS	dPCS	N.A.	Total
Subject 01	2 (18%)	1 (9%)	2 (18%)	2 (18%)	2 (18%)	2 (18%)	0 (0%)	11
Subject 02	4 (36%)	2 (18%)	1 (9%)	1 (9%)	1 (9%)	2 (18%)	0 (0%)	11
Subject 03	2 (18%)	3 (27%)	1 (9%)	1 (9%)	3 (27%)	1 (9%)	0 (0%)	11
Subject 04	5 (45%)	1 (9%)	1 (9%)	1 (9%)	0 (0%)	2 (18%)	1 (9%)	11
Subject 05	3 (25%)	4 (33%)	1 (8%)	2 (17%)	0 (0%)	0 (0%)	2 (17%)	12
Subject 06	2 (18%)	2 (18%)	2 (18%)	4 (36%)	1 (9%)	0 (0%)	0 (0%)	11
Subject 07	2 (18%)	1 (9%)	1 (9%)	2 (18%)	3 (27%)	1 (9%)	1 (9%)	11
Subject 08	4 (29%)	1 (7%)	4 (29%)	3 (21%)	1 (7%)	1 (7%)	0 (0%)	14
Subject 09	4 (29%)	3 (21%)	0 (0%)	1 (7%)	2 (14%)	2 (14%)	2 (14%)	14
Subject 10	3 (30%)	2 (20%)	0 (0%)	2 (20%)	0 (0%)	1 (10%)	2 (20%)	10
Subject 11	2 (22%)	3 (33%)	1 (11%)	1 (11%)	1 (11%)	1 (11%)	0 (0%)	9
Subject 12	2 (17%)	2 (17%)	1 (8%)	0 (0%)	1 (8%)	0 (0%)	6 (50%)	12
Subject 13	5 (38%)	2 (15%)	2 (15%)	1 (8%)	0 (0%)	1 (8%)	2 (15%)	13
Subject 14	2 (20%)	3 (30%)	1 (10%)	1 (10%)	1 (10%)	2 (20%)	0 (0%)	10
Subject 15	1 (9%)	2 (18%)	1 (9%)	1 (9%)	1 (9%)	3 (27%)	2 (18%)	11
Subject 16	4 (40%)	1 (10%)	2 (20%)	1 (10%)	2 (20%)	0 (0%)	0 (0%)	10
Subject 17	4 (40%)	5 (50%)	0 (0%)	0 (0%)	0 (0%)	0 (0%)	1 (10%)	10
Subject 18	3 (27%)	2 (18%)	0 (0%)	1 (9%)	3 (27%)	2 (18%)	0 (0%)	11
Subject 19	1 (9%)	3 (27%)	1 (9%)	1 (9%)	1 (9%)	3 (27%)	1 (9%)	11
Subject 20	3 (23%)	3 (23%)	2 (15%)	2 (15%)	1 (8%)	2 (15%)	0 (0%)	13
Proportion	26%	20%	11%	12%	11%	12%	9%	
Presence	20/20	20/20	16/20	18/20	15/20	15/20		

N.A.: not assigned.

**Figure 5:**
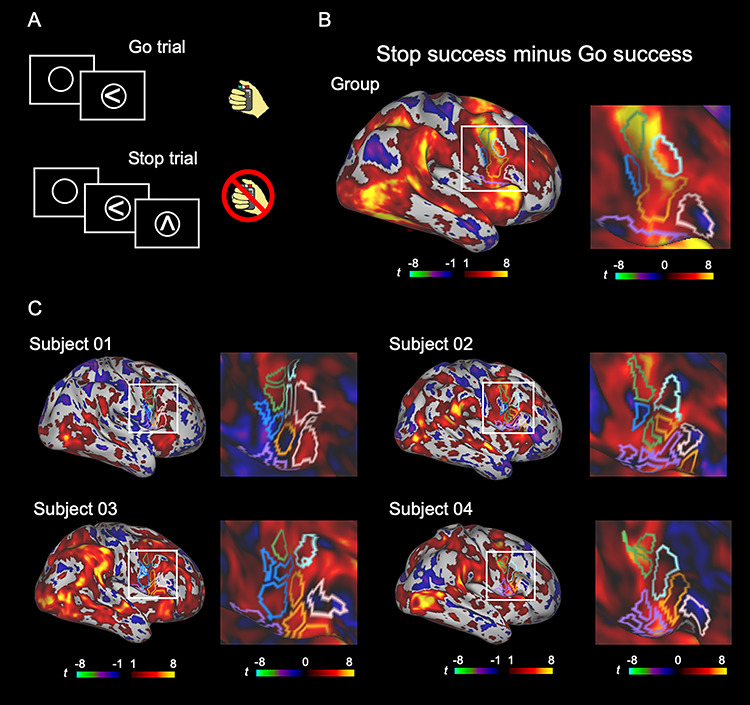
The vertex-wise brain activity during response inhibition. (*A*) The basic structure of the stop-signal task. (*B*, *C*) Vertex-wise brain activity maps in the group (*B*) and in the four representative subjects (*C*) to inspect the spatial extent of the brain activity. A general linear model was applied to each vertex, and Stop success and Go success trials were contrasted. The color scales indicate *t* values in the vertices.

The subjects performed the stop-signal task in the scanner (Fig. 5*A*). Table 2 summarizes the behavioral data. Reaction time for Go success trials was significantly longer than that for Stop failure trials (*t*(19) = 9.6, *P* < 0.001, paired *t*-test). The reaction time for Stop failure trials with longer SSDs was significantly longer than that for Stop failure trials with shorter SSDs (*t*(19) = 12.1, *P* < 0.001, paired *t*-test). Supplementary Figure 2 shows the normalized inhibition function that were used to plot the proportion of Stop failure trials as a function of the relative finishing times (RFT) of the response and stop processes. Z-transformed RFT (ZRFT) was calculated as ZRFT = (mean[RTgo] − SSD − SSRT)/SD[RTgo]. Positive/negative ZRFT values indicate shorter/longer SSD. These results confirmed that the assumptions of the independent race model were fulfilled.

**Table 2:** Behavioral data for the stop-signal task

Reaction time [Go success] (ms)	457.7 ± 52.4
SSD (ms)	237.1 ± 62.9
SSRT (ms)	208.9 ± 31.7
Correct response rate [Go] (%)	98.7 ± 1.9
Correct response rate [Stop] (%)	49.9 ± 1.6
Reaction time [Stop failure] (ms)	426.1 ± 50.9
Reaction time [Stop failure|shorter SSDs] (ms)	395.5 ± 50.0
Reaction time [Stop failure|longer SSDs] (ms)	457.1 ± 56.7

Behavioral data for the stop-signal task under fMRI scanning (mean ± SD). Shorter/longer SSDs indicate trials with shorter/longer SSDs than median SSD.

Figure 5*B* shows the vertex-wise brain activity during response inhibition (contrast: Stop success minus Go success) at the group level, overlaid with the boundaries of the IFC parcels assigned to the six modules. The brain activity and its correlation with behavior in the whole brain were shown in Supplementary Figure 3. The brain activity and its correlation with behavior were further calculated on the parcel basis using the parcels of Gordon et al. (2016) (Supplementary Fig. 4). Figure 5*C* shows the vertex-wise brain activity during response inhibition at the individual level (see Supplementary Fig. 5 for all the subjects). The boundaries of the mIFC parcels of individual subjects appeared to demonstrate moderate correspondence with the spatial extent of the low level of brain activity, as seen in the group results.

**Figure 6:**
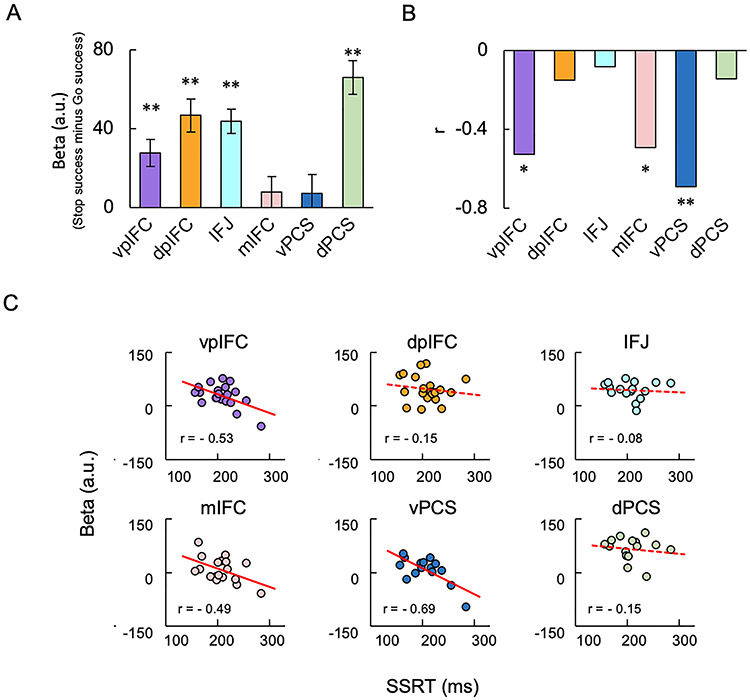
Brain activity and brain-behavior correlation in six modules during response inhibition. (*A*) The brain activity in the six modules in the right IFC averaged across subjects. Error bars indicate the standard error of means of the subjects. ^*^^*^P < 0.01, one-sample *t*-test. (*B*) The correlation between the brain activity and SSRT in the six modules. ^*^P < 0.05; ^*^^*^P < 0.01. (*C*) The scatter plots of the correlation in the six modules.

The brain activity was quantified in the six modules of individual subjects (Fig. 6*A*). The brain activation was prominent in the parcels near the anterior bank of the precentral sulcus, the vpIFC module (*t*(19) = 4.0, *P* < 0.001, one sample *t*-test), dpIFC module (*t*(19) = 5.6, *P* < 0.001, one sample *t*-test), IFJ module (*t*(19) = 7.2, *P* < 0.001, one sample *t*-test), and dPCS module (*t*(19) = 7.8, *P* < 0.001, one sample *t*-test). On the other hand, the correlation between the brain activity and SSRT was significant in the vpIFC module (*r* = −0.53, *P* = 0.02), mIFC module (*r* = −0.49, *P* = 0.04), and vPCS module (*r* = −0.69, *P* = 0.004) (Fig. 6*B*,*C*). As a control, the brain activity and its correlation with SSRT were calculated by applying common group-level parcels to all the subjects. As shown in Supplementary Figure 6, the significance levels of the correlation appear less differentiated when the group-level parcels were used. For example, the correlations with SSRT in the dpIFC, mIFC, and vPCS, which were statistically mediocre in the group-level parcels, were almost absent in the dpIFC and statistically significant in the mIFC and vPCS in the individual-focused approach (Fig. 6). Since the significance level of the correlations in the individual-focused approach was marginal, an independent dataset from our previous study of the stop-signal task using larger samples (*N* = 46) (Jimura et al. 2014) was used to replicate the results. Each vertex in the six group-level parcels in the IFC was mapped to a three-dimensional voxel in MNI space in the dataset, and correlation between the brain activity and SSRT was calculated in each corresponding parcel. The correlations were significant in the vpIFC, mIFC, and vPCS (vpIFC: *r* = −0.37, *P* = 0.01; mIFC: *r* = −0.38, *P* = 0.01; and vPCS: *r* = −0.30, *P* = 0.04) but not significant in the dpIFC, IFJ, and dPCS (dpIFC: *r* = −0.19, *P* = 0.2; IFJ: *r* = −0.23, *P* = 0.1; and dPCS: *r* = −0.26, *P* = 0.08).

We also performed network analyses, assigning the 17 known networks in the whole cerebral cortex of individual brains using Infomap (Rosvall and Bergstrom 2008) following Power et al. (2011) and Gordon et al. (2017b) (Supplementary Fig. 7*A*). We then compared the six modules with the 17 networks in the right IFC of individual subjects. Supplementary Figure 7*B* shows the percentage of the 17 networks located in each of the six modules in the right IFC. Most of the parcels assigned as the vpIFC module belonged to the cingulo-opercular network. Most of the parcels assigned as the dpIFC or IFJ module belonged to the frontoparietal network, consistent with the high similarity of connectivity between the dpIFC and IFJ modules. Most of the parcels in the mIFC, vPCS, and dPCS modules belonged to the default mode, dorsal attention, and cingulo-opercular network, respectively. The brain activity and its correlation with behavior were further quantified for each module on the basis of the parcels belonging to the single network. The brain activity in each module that belonged to the dominant network is shown in Supplementary Figure 7*C*, and its correlation with behavior is shown in Supplementary Figure 7*D*,*E*. The results appeared basically similar to those presented in Figure 6.

## Discussion

In the present functional MRI study, precision functional mapping of individual human brains was adopted to the parcels in the right IFC for a more comprehensive and precise understanding of the areal organization of the right IFC. The functional properties of six modules during response inhibition were evaluated. The areal organization of the right IFC varied considerably across subjects, and the six modules in the right IFC showed distinct patterns of characteristics in the brain activity and its correlation to behavior. These results provide finer details of the functional features of the IFC parcels and highlight the utility of the precision brain mapping approaches in neuroimaging and neuromodulation studies of response inhibition.

The number of parcels in the whole cerebral cortex at the group level in the present study was 377, consistent with those in previous studies of cortical parcellation: 268 in Finn et al. (2015), 360 in Glasser et al. (2016), 333 in Gordon et al. (2016), and 400 in Schaefer et al. (2018). The average number of cortical parcels of individual brains, on the other hand, was 612 in the present study, whereas those in previous studies were 616 in Laumann et al. (2015) and 621 in Gordon et al. (2017b). Thus, the number of cortical parcels of individual brains was approximately double of that at the group level, which allowed us to evaluate the variability of the location and extent of the six modules in a parcel-wise fashion. Whereas prior studies employing the precision mapping approach have investigated the individual variability of cerebrocortical networks (Mueller et al. 2013; Harrison et al. 2015; Laumann et al. 2015; Wang et al. 2015; Braga and Buckner 2017; Gordon et al. 2017a, 2017b; Gratton et al. 2018; Kong et al. 2019; Seitzman et al. 2019), the present study focused on the variability of the right IFC at the parcel level to investigate more local functional properties of the IFC parcels.

The advantage of the individual-focused approach may be seen in the results of the present study. For example, the correlations in the dpIFC, mIFC, and vPCS were not significant in the group-level parcels (Supplementary Fig. 6). In the individual-focused approach, the correlations in the mIFC and vPCS were significant, and the correlation in the dpIFC was nearly absent, as presented in Figure 6. The comparison between the group-level and individual-focused approaches suggests that the latter approach sharpens the significance level of the correlation between the brain activity and SSRT. It is to be noted that parcels in the same module sometimes appear to be “split” in a noncontiguous manner. Such phenomena can be seen in highly sampled subjects (Gordon et al. 2017b), suggesting that the individual-focused approach can detect small, spatially variable module pieces of individual brains.

The IFC parcels of individual brains were assigned to the six modules in the present study, and the same parcels were also assigned to the 17 networks using Infomap-based community detection analysis (Supplementary Fig. 7). Most of the IFC parcels in each module were assigned to a common particular network. It should be noted that most of the parcels assigned to the dpIFC and IFJ modules commonly belonged to the frontoparietal network, although the second top networks were different. The results denied a simple view that the six modules corresponded to six (out of 17) different particular networks. One likely explanation for the discrepancy would be that the six modules were not located in the centers of the 17 network clusters (Gordon et al. 2017b) in the spring-embedded plots showing the functional connectivity profiles of the whole cortical parcels (Power et al. 2011). The missing modules in some subjects (the IFJ, mIFC, vPCS, and dPCS modules) may also be explained by interindividual variability in the network configuration (Gordon et al. 2017b), which may have led to atypical configuration of the IFC modules.

Brain activation during response inhibition has been reported in cortical and subcortical regions including the IFC, presupplementary motor area (preSMA), intraparietal sulcus (IPS), and temporoparietal junction (TPJ) (Aron and Poldrack 2006; Chikazoe et al. 2009; Neubert et al. 2010; Sharp et al. 2010; Zandbelt and Vink 2010; Whelan et al. 2012; Cai et al. 2014; Erika-Florence et al. 2014; Rae et al. 2015; Watanabe et al. 2015; Yamasaki et al. 2018; Osada et al. 2019). In the IFC, the use of the individual-focused approach in the present study showed that significant brain activity during response inhibition was observed in rather limited regions: the vpIFC, dpIFC, IFJ, and dPCS modules, centered around the anterior bank of the precentral sulcus. Along with other regions outside the IFC showing the correlation between the brain activity and SSRT (Li et al. 2006, 2008; Aron et al. 2007; Forstmann et al. 2008; Hirose et al. 2012a), significant correlations were also observed in the IFC regions in the present study: the vpIFC, mIFC, and vPCS modules. The dpIFC, IFJ, and dPCS were active but were not associated with performance, and the mIFC and vPCS were not active but were associated with performance. These two types of regions were both thought to be related to response inhibition, but the functional difference was not clearly understood. A significant correlation has been reported in a region significantly activated during response inhibition (Aron et al. 2007), which is likely located in the vpIFC module that showed both significant brain activity and its correlation with behavior. Regions that showed significant brain activity, the dpIFC and IFJ modules, have also been highlighted in response inhibition (Derrfuss et al. 2005; Chikazoe et al. 2009). A significant correlation was also reported in a region that is not activated during response inhibition (Jimura et al. 2014), which is likely located in the mIFC module. Although there have been few studies, to our knowledge, that highlighted the posterior bank of the precentral sulcus, the significant brain activity in dPCS module and the significant correlation in the vPCS module may raise the possibility that they play important roles for response inhibition. More broadly, the precision functional mapping at the single-subject level may raise important hypotheses to be tested for other cognitive functions in local regions in the cerebral cortex (Miyashita 2016).

## Funding

JSPS KAKENHI (Grant Number 18K07348 to T.O., 19K07807 to A.O.); Takeda Science Foundation (to S.K.).

## Notes

We thank Mr T. Kamiya and Mr H. Goto for technical assistance. *Conflict of Interest*: None declared.

## Supplementary Material

SFig1_bhaa188

SFig2_bhaa188

SFig3_bhaa188

SFig4_bhaa188

SFig5_bhaa188

SFig6_bhaa188

SFig7_bhaa188

SupTable1_bhaa188

SupTable2_bhaa188

Manuscript2_supp_legends_bhaa188
