# Supplementary figures and images for: Functional Organization for Response Inhibition in the Right Inferior Frontal Cortex of Individual Human Brains
Source: Cereb Cortex. 2020 Jul 15;30(12):6325–35. doi: 10.1093/cercor/bhaa188 (PMC7609925; doi:10.1093/cercor/bhaa188)

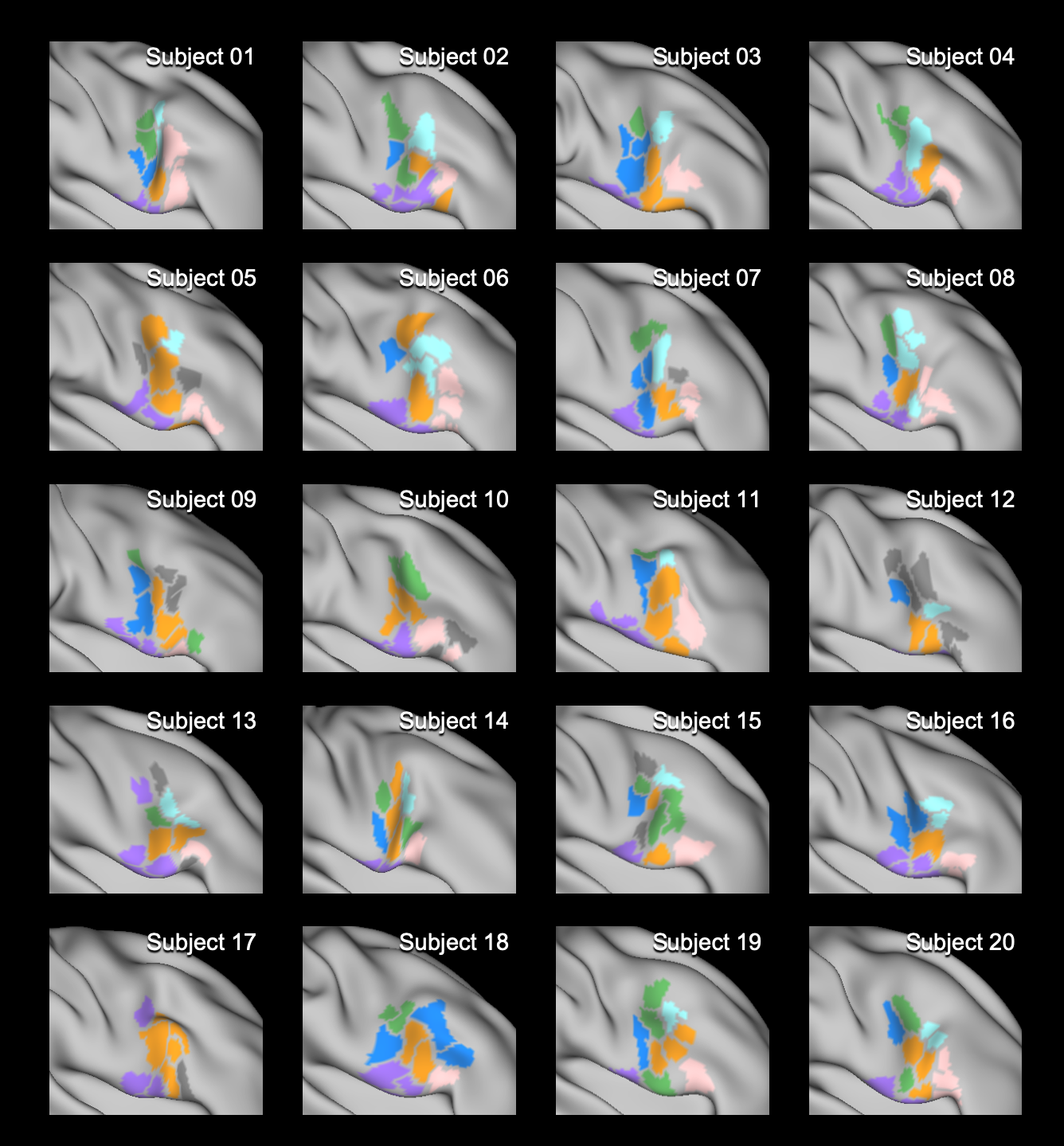

Supplement: SFig1_bhaa188 [file sfig1_bhaa188.png]

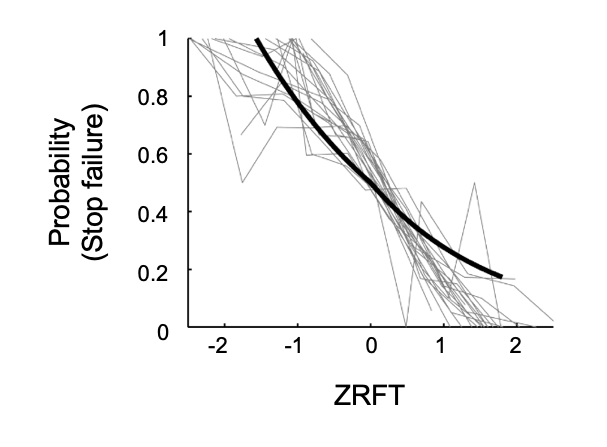

Supplement: SFig2_bhaa188 [file sfig2_bhaa188.png]

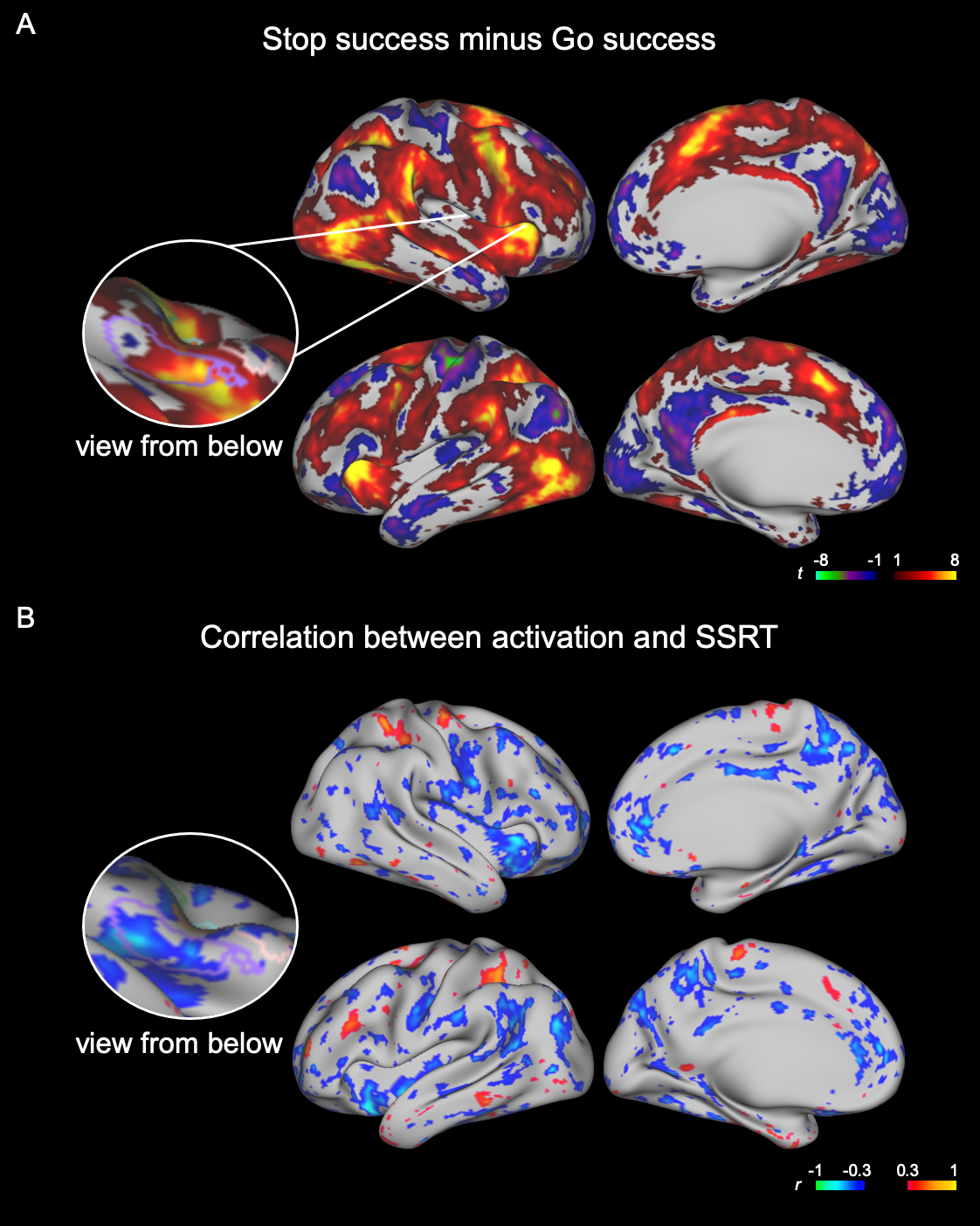

Supplement: SFig3_bhaa188 [file sfig3_bhaa188.png]

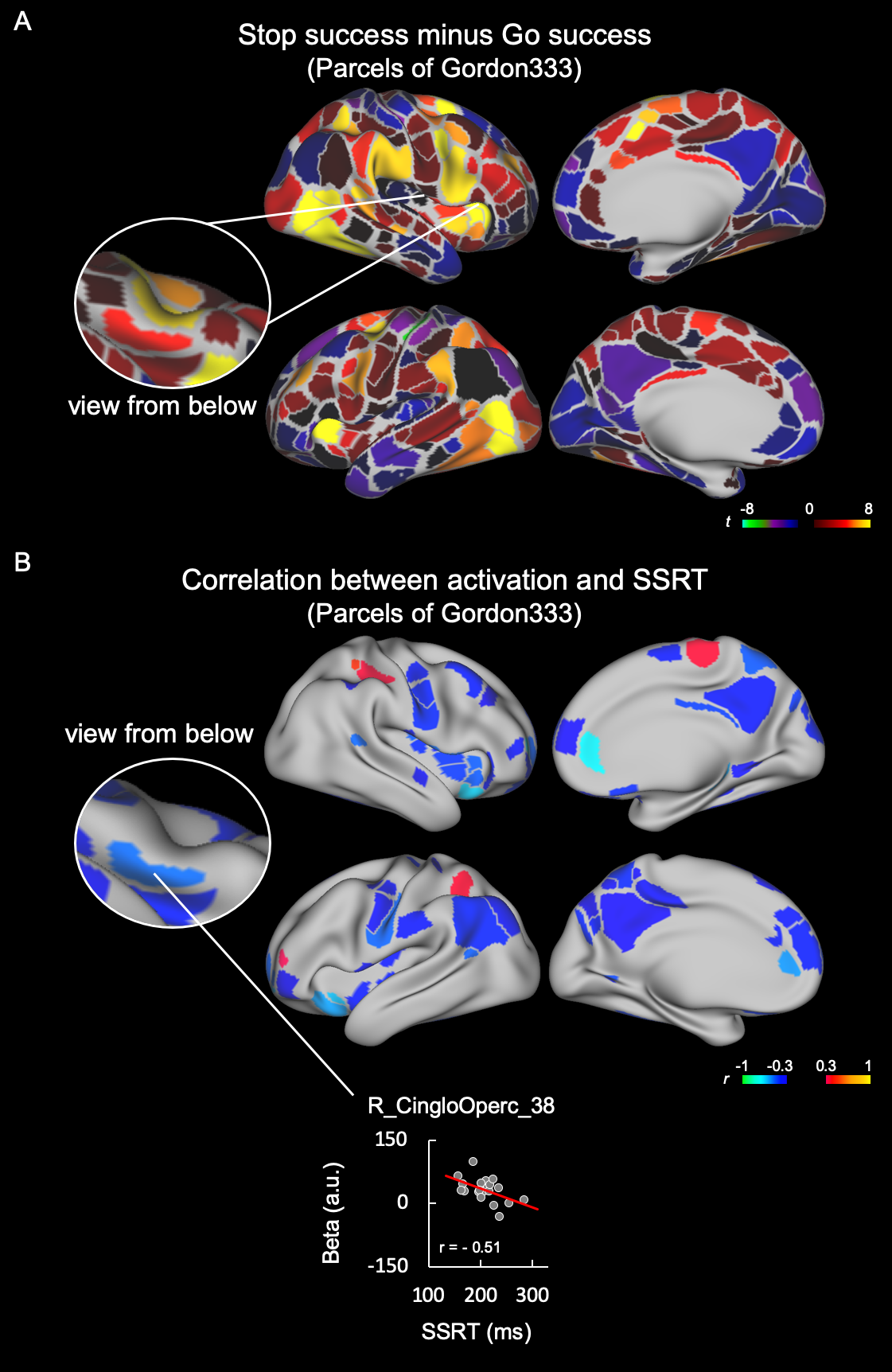

Supplement: SFig4_bhaa188 [file sfig4_bhaa188.png]

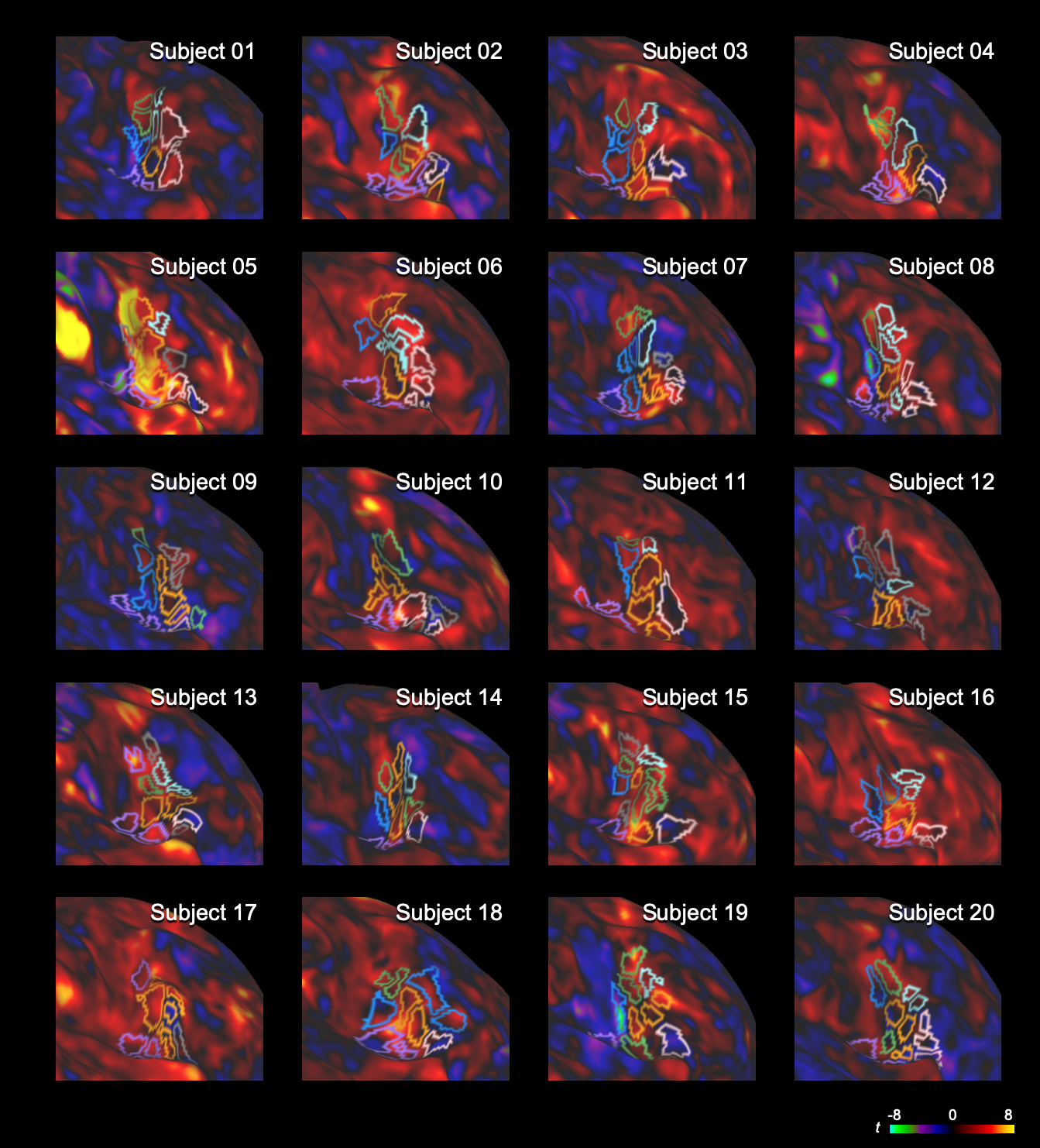

Supplement: SFig5_bhaa188 [file sfig5_bhaa188.png]

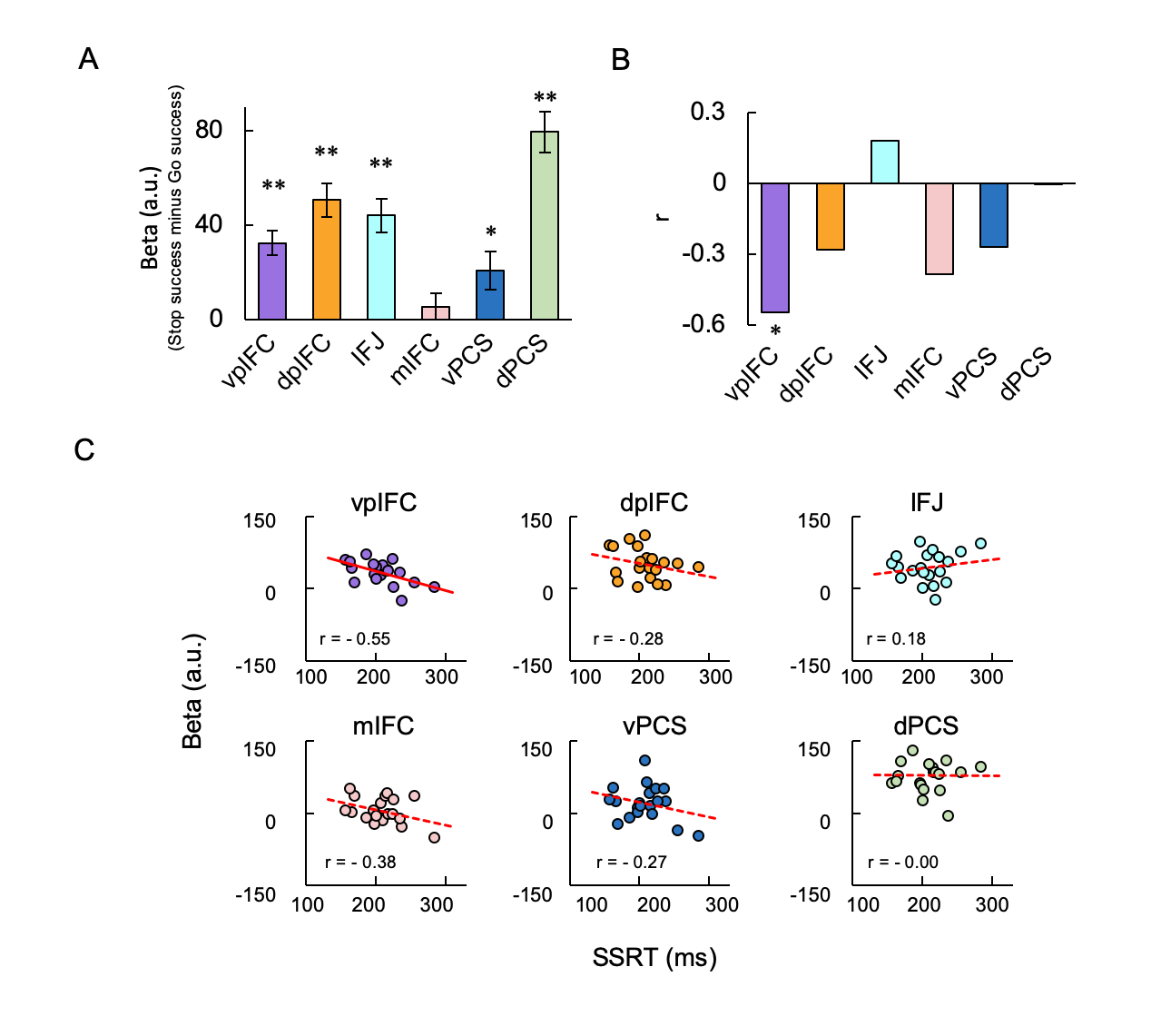

Supplement: SFig6_bhaa188 [file sfig6_bhaa188.png]

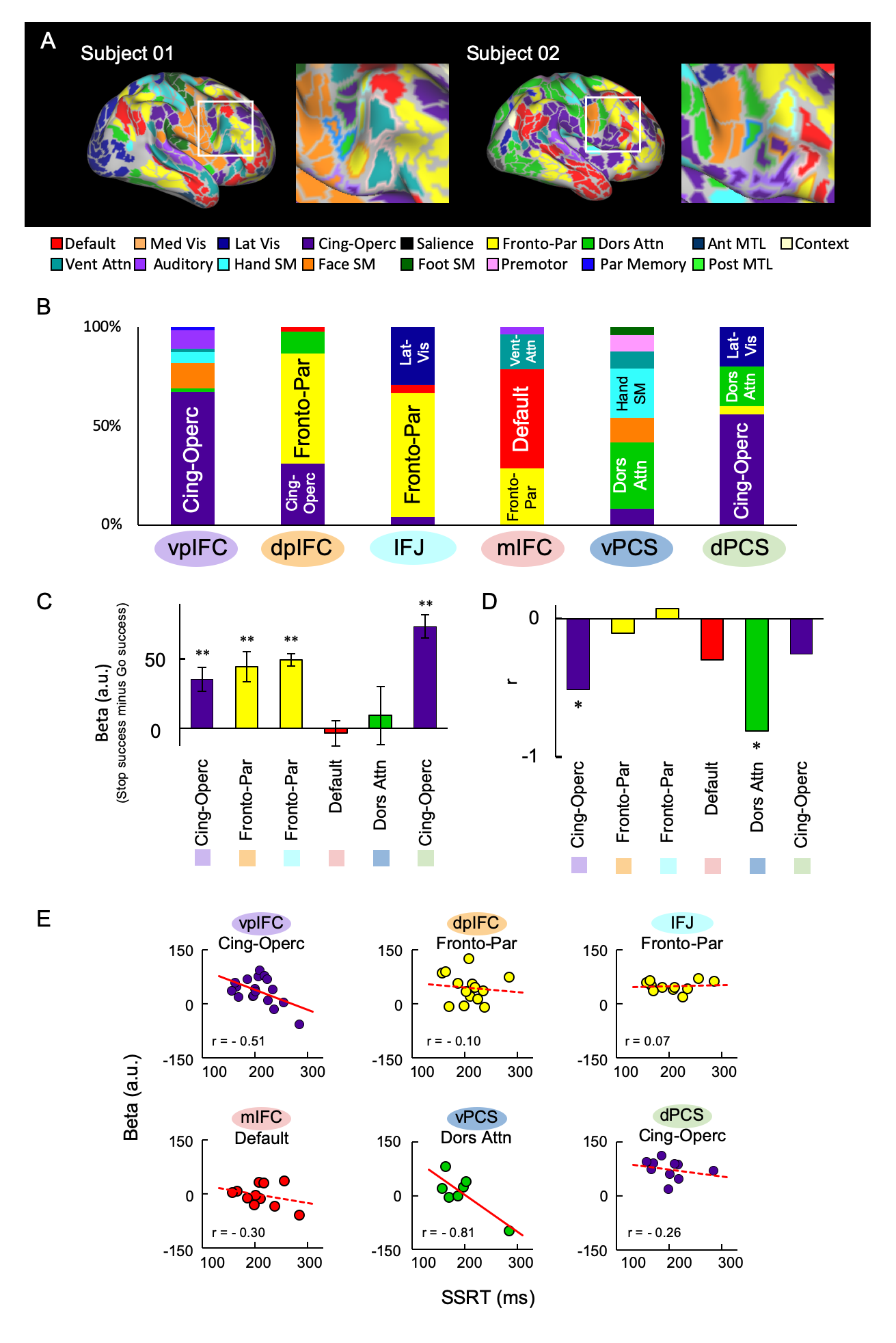

Supplement: SFig7_bhaa188 [file sfig7_bhaa188.png]
