# Supplementary material for: Functional Organization for Response Inhibition in the Right Inferior Frontal Cortex of Individual Human Brains
Source: Cereb Cortex. 2020 Jul 15;30(12):6325–35. doi: 10.1093/cercor/bhaa188 (PMC7609925; doi:10.1093/cercor/bhaa188)
Supplement: SupTable1_bhaa188 [file suptable1_bhaa188.pdf]

Supplementary Table 1. MNI coordinates of the centroids of group-level IFC parcels.

vpIFC	[55, 7, 1]
dpIFC	[45, 10, 20]
IFJ	[34, 11, 29]
mIFC	[56, 26, 16]
vPCS	[55, 9, 28]
dPCS	[44, 2, 36]
