# Supplementary material for: Functional Organization for Response Inhibition in the Right Inferior Frontal Cortex of Individual Human Brains
Source: Cereb Cortex. 2020 Jul 15;30(12):6325–35. doi: 10.1093/cercor/bhaa188 (PMC7609925; doi:10.1093/cercor/bhaa188)
Supplement: SupTable2_bhaa188 [file suptable2_bhaa188.pdf]

Supplementary Table 2. Classifications of the six group-level parcels according to the previous studies.

	Schaefer et al., 2018 (300)	Glasser et al., 2016 (360)	Gordon et al., 2016 (333)	Finn et al., 2015 (268)	Shen et al., 2013 (278)	Craddock et al., 2012 (300)	Bellec et al., 2010 (325)
**vpIFC**	SalVentAttnA_FrOper_1	R_FOP1	R_CinguloOperc_38	61 Motor	R.BA44.2	124	218
**dpIFC**	ContA_PFCl_1/3	R_6r	R_DorsalAttn_32	21 Frontoparietal	R.BA44.2	161	319
**IFJ**	ContA_PFCl_4	R_IFJp	R_DorsalAttn_32	22 Frontoparietal	R.BA44.1	39	319
**mIFC**	DefaultB_PFCv_3	R_44/45	R_VentralAttn_18	16 Medial frontal	R.BA47.4	280	300
**vPCS**	ContA_PFCl_3	R_6v	R_DorsalAttn_32	21 Frontoparietal	R.BA6.9	148	319
**dPCS**	SalVentAttnA_PrC_1	R_PEF	R_DorsalAttn_31	31 Frontoparietal	R.BA6.6	39	319

(total number of parcels)
