# Supplementary material for: Functional Organization for Response Inhibition in the Right Inferior Frontal Cortex of Individual Human Brains
Source: Cereb Cortex. 2020 Jul 15;30(12):6325–35. doi: 10.1093/cercor/bhaa188 (PMC7609925; doi:10.1093/cercor/bhaa188)
Supplement: Manuscript2_supp_legends_bhaa188 [file manuscript2_supp_legends_bhaa188.docx]

**Supplementary Figure 1.**

The right IFC parcels assigned to the six modules in all the subjects. The format is similar to Fig. 4A.

**Supplementary Figure 2.**

The normalized inhibition function that plots the proportion of Stop failure trials as a function of the relative finishing times (RFT) of the response and stop processes. Z-transformed RFT (ZRFT) was calculated as ZRFT = (Mean[RTgo] – SSD – SSRT)/SD[RTgo].

**Supplementary Figure 3.**

(A) Vertex-wise brain activity maps during response inhibition in the whole brain at the group level. A purple line delineates the vpIFC in the group-level parcels. (B) Vertex-wise correlation maps between the brain activity and SSRT.

**Supplementary Figure 4.**

Brain activity maps (A) and correlation maps with behavior (B) calculated on the parcel basis using the 333 parcels of Gordon et al. (2016). The vpIFC in the group-level parcels of the present study corresponds to R_CinguloOperc_38 in Gordon et al. (2016).

**Supplementary Figure 5.**

Vertex-wise brain activity maps during response inhibition for all individual subjects.

**Supplementary Figure 6.**

(A) The brain activity in the six group-level parcels in the right IFC averaged across subjects. Error bars indicate the standard error of means of the subjects. *p < 0.05; **p < 0.01, one-sample t-test. (B) The correlation between the brain activity and SSRT in the group-level six parcels. *p < 0.05. (C) The scatter plots of the correlation.

**Supplementary Figure 7.**

(A) Assignment of the 17 known networks in the whole cerebral cortex of individual brains of the two representative subjects using Infomap. (B) The percentage of the 17 networks located in each of the six modules in the IFC. (C) The brain activity in each module that belonged to the dominant network averaged across subjects. Error bars indicate the standard error of means of the subjects. **p < 0.01, one-sample t-test. (D) The correlation between the brain activity and SSRT. *p < 0.05. (E) The scatter plots of the correlation.
